# Supplementary figures and images for: Genome-Wide Identification of SMXL Gene Family in Soybean and Expression Analysis of GmSMXLs under Shade Stress
Source: Plants (Basel). 2022 Sep 15;11(18):2410. doi: 10.3390/plants11182410 (PMC9500757; doi:10.3390/plants11182410)

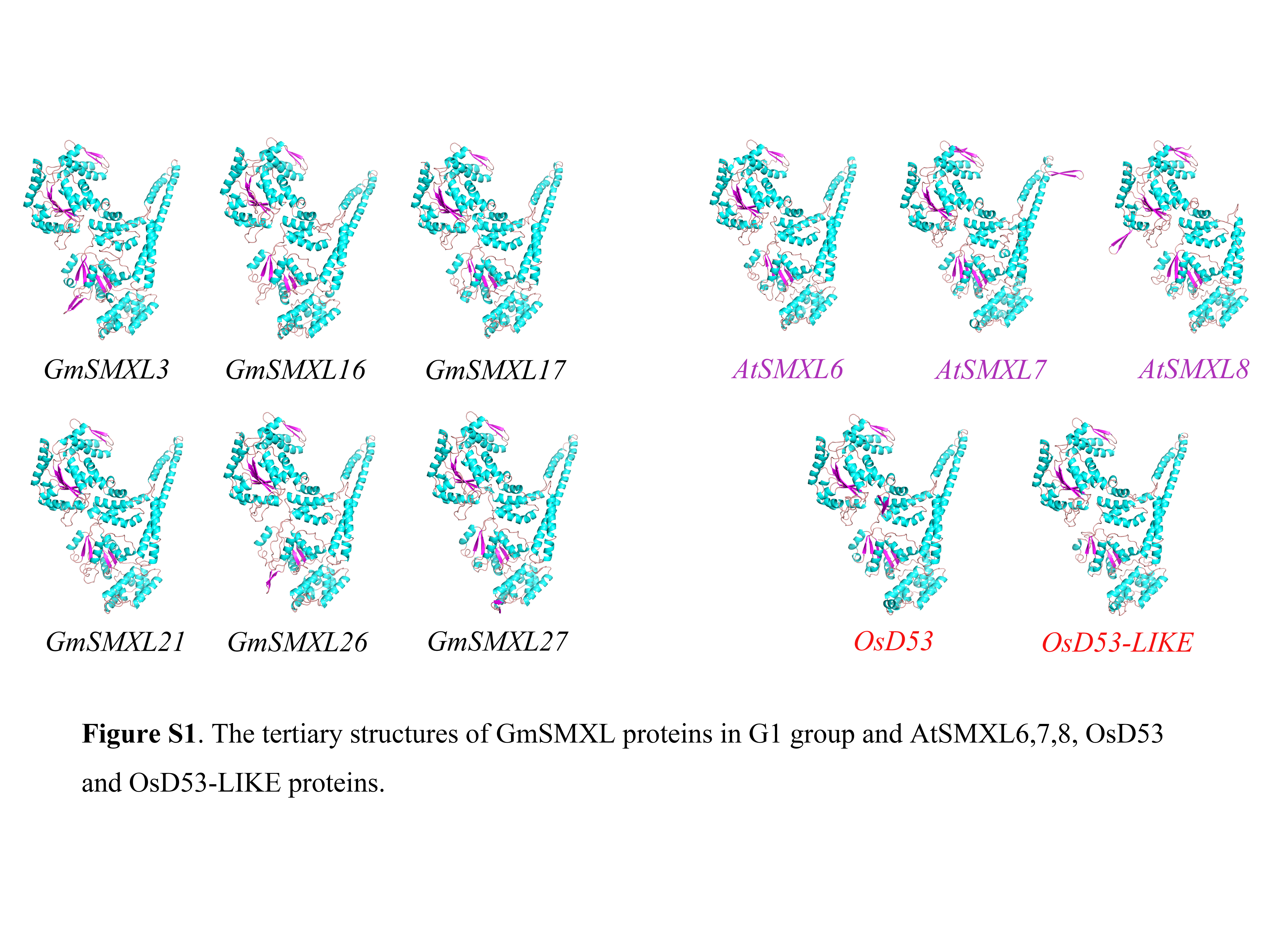

Supplement: Supplementary file 1 [file plants-11-02410-s001.zip › Figure S1. The tertiary structures of GmSMXL proteins in G1 group and AtSMXL6,7,8, OsD53 and OsD53-LIKE proteins.jpg]
